# Effects of Matcha Green Tea Powder on Cognitive Functions of Community-Dwelling Elderly Individuals

**DOI:** 10.3390/nu12123639

**Published:** 2020-11-26

**Authors:** Keisuke Sakurai, Chutong Shen, Yuri Ezaki, Noriko Inamura, Yoichi Fukushima, Nobutaka Masuoka, Tatsuhiro Hisatsune

**Affiliations:** 1 Department of Integrated Biosciences, Graduate School of Frontier Sciences, The University of Tokyo, Kashiwa 277-8562, Japan; 3647306978@edu.k.u-tokyo.ac.jp (K.S.); qazwszmxn@gmail.com (C.S.); yuri8211@gmail.com (Y.E.); nbtkmska@gmail.com (N.M.); 2 Community Health Promotion Laboratory, Mitsui Fudosan, Co., Ltd., Kashiwa 277-8519, Japan; inamura@udck.jp; 3 Urban Design Center Kashiwanoha (UDCK), Kashiwa 277-0871, Japan; 4 Marketing & Communications Division, Nestle Japan Ltd., Tokyo 140-0002, Japan; Yoichi.Fukushima@jp.nestle.com

**Keywords:** green tea, cognitive function, memory function, impulsivity, aging, vitamin K

## Abstract

Matcha Green Tea Powder contains a variety of active ingredients beneficial to health, such as tea catechins, lutein and vitamin K. It is also known that these ingredients confer benefits upon cognitive functions of elderly people. Therefore, we aimed to investigate the relationship between a daily supplementation of Matcha and the change in cognitive functions of community-dwelling elderly people. A randomized, double-blind, placebo-controlled 12-week trial was performed. Sixty-one participants were recruited and randomly assigned to receive test drink containing 3 g powder from fresh Matcha or placebo powder per day. Changes in cognitive function were assessed utilizing a psychometric test battery. Daily food intake was assessed by a Brief-type Self-administered Diet History Questionnaire (BDHQ). In the gender-specific analysis, a significant cognitive enhancement was observed in the Montreal Cognitive Assessment (MoCA) score in the active group of women. In dietary analysis, we found a significant inverse correlation between consumption of vitamin K in daily diet, excluding test drinks, and change in MoCA. The present study suggests that daily supplementation of Matcha Green Tea Powder has protective effects against cognitive decline in community-dwelling elderly women.

## 1. Introduction

Tea (*Camellia sinensis*) is one of the most popular drinks in the world. In fact, the green tea market is growing worldwide, and green tea production in 2023 is expected to be double that of 10 years ago [1]. Many studies have shown various health benefits of tea consumption, such as reducing the risk of stroke [2,3] by lowering blood pressure [4,5], improving psychopathological symptoms and providing neuroprotection [6]. In particular, green tea consumption is said to have a positive effect on stress relief and anxiety reduction in individuals who already suffer from psychopathological symptoms [7,8]. Furthermore, green tea has improved memory and attention [8,9], and activated working memory seen in functional magnetic resonance imaging (MRI) [10,11]. As these health benefits of drinking green tea are more widely known, not only the consumption of tea itself but the number of people taking tea extracts as a supplement is increasing, and the intake of tea and its bioactive constituents has been recommended for health [12,13].

On the other hand, the risk of dementia increases with aging, and dementia is the second leading cause of death over 70 [14]. Alzheimer’s disease (AD) is the most common cause of dementia, accounting for about 70% [15]. AD progresses gradually through mild cognitive impairment (MCI), which can be referred to as the predementia phase of AD [16]. The conversion rate from MCI to dementia is reported to be about 10% per year, while the reversion rate is about 24% [17,18]. Therefore, in order to reduce the number of AD patients in the future, intervention in patients with normal or with mild cognitive symptoms is necessary. At present, however, there exists no pharmacological treatment that has been established to be effective in preventing people with normal cognitive function or MCI from progressing to dementia [19]. Thus, intervention in these people’s daily habits is one of the few options available to reduce the onset of AD, and nutritional intervention is considered to be one of these [20]. In fact, our randomized controlled trial in the laboratory has shown that the nutritional supplementation of anserine and carnosine in healthy elderly people and MCI patients helped them maintain cognitive functions [21,22,23]. While cognitive functions and memory decline during the progression of MCI and AD, impulsivity is known to increase [24,25,26]. In AD patients, elevated impulsivity is observed as one of the behavioral and psychological symptoms of dementia (BPSD) and is a heavy burden for caregivers [27]. However, impulse control disorder is observed not only in AD but also as a symptom of other types of dementia such as Frontotemporal Dementia [28,29]. Impulsivity is a facet of personality and has a multidimensional structure. According to Moller et al., it is defined as “predisposition toward rapid, unplanned reactions to internal or external stimuli without regard to the negative consequences of these reactions to the impulsive individual or to others [30]”. However, impulsivity is an ambiguous concept and covers a wide range of “actions that are poorly conceived, prematurely expressed, unduly risky, or inappropriate to the situation and that often result in undesirable outcomes [31]”. Besides, impulsivity is an intermediate phenotype of behavioral abnormalities and elevated impulsivity can cause a variety of social problems such as substance abuse [32], gambling addiction [33] and overeating [34]. Several personality tests have been developed to evaluate impulsivity, of which the Barratt Impulsiveness Scale-11 (BS-11) is the most widely used impulsivity evaluation test. BIS-11 has been translated into various languages and used all over the world. The Japanese version of BIS-11 was translated by Kobashi and Ida. The underlying mechanisms of impulsivity have not been well documented [35]. Especially, although green tea has been reported to have some positive effects on mental health such as reduction of anxiety [36], its effects on impulsivity have not been investigated at all. Therefore, further research is necessary.

Green tea provides many groups of nutrients, including polyphenols, amino acids, and some trace elements [37]. Matcha, a special type of green tea, forms part of the traditional Japanese tea culture. Although Matcha is derived from the same *Camellia sinensis* plant as normal green tea, Matcha is cultivated and processed differently. First, during the cultivation, green tea is grown in the sun; however, Matcha is grown under shade during the final few weeks before harvest. Therefore, this different cultivating leads to the higher amount of tea theanine contained inside Matcha green tea, which gives it a special taste called “Umami” that may balance the usual bitter taste of tea. What is more, the processing of normal green tea and Matcha are also different. When processing normal green tea, the procedure usually includes sun-drying, tumbling and steaming. However, during the processing of Matcha green tea, tea leaves are destemmed and deveined, and only steamed shortly after harvest. Therefore, due to this special processing, Matcha green tea has a bright green color, and the nutrient loss during the steaming process in normal green tea can also be prevented and may lead to much more health benefits. Besides, because of the higher “Umami” which can be tasted in Matcha, this may balance the bitter taste derived from catechin, and improve the palatability of the tea. In other words, due to the usual bitter taste derived from catechin, regular green tea powder on the market can only contain 0.5 g of tea leaves per serving, or the bitter taste will make it unacceptable. However, the “Umami” taste derived from theanine inside Matcha may weaken the bitter taste, and increase palatability, which make it possible to have 1.5 g tea leaves per serving in Matcha green powder product. Thus, due to several special characteristics of Matcha green tea, it is suggested that drinking the same serving provides a better taste than regular green tea, and also provides twice the amount of polyphenols and higher fat-soluble nutrients such as Vitamin K and lutein in each single serving. Notably, some of these nutrients are said to aid in cognitive function. The anti-inflammatory and strong antioxidant properties of epigallocatechin gallate (EGCG), a famous polyphenol and one of the most abundant catechins inside green tea extract, is widely known [38,39], and it is also suggested that it acts as an active compound that ameliorates cognitive defects [40]. L-theanine, the main amino acid in tea, is also said to ease the symptoms of cognitive impairment and assisted in the hippocampal long-term potentiation of Alzheimer’s disease in mice [41]. Its role in anxiety and stress relief and improving sleep quality is commonly known [42,43], and it has been suggested to possess the ability to prevent stress-induced brain atrophy by modifying early stress responses [44]. Caffeine, which is abundant in tea, strengthens the effect of theanine on the enhancement of neurophysiological performance, such as attention [45]. As for fat-soluble nutrients, the role of vitamin K and lutein has been well noted. Vitamin K, while it is more commonly known for its important role in blood coagulation [46], also acts as an essential nutrient in the central nervous system (CNS) [47,48]. Previous evidence has also shown that higher serum phylloquinone status in elderly people has been associated with better performance in verbal episodic memory, and higher dietary intake of vitamin K was also linked with less severe subjective memory complaints among older adults [49,50]. On the other hand, being a kind of carotenoid, lutein comprises not only the ability to reduce the risk of some chronic health disorders, but is also believed to aid in cognitive function, and is associated with word recall ability among older adults [51,52]. Based on these nutritional facts, we aimed to use a Matcha green tea extract to investigate the effects on cognitive memory function and impulsivity in clinically normal elderly people. In this study, we conducted a randomized, double-blind, placebo-controlled 12 weeks trial using a psychometric test battery, and secondarily we also investigated the difference in effect by gender.

## 2. Materials and Methods

### 2.1. Study Design and Participants 

This study was a randomized, double-blind, placebo-controlled trial, which aimed to evaluate the efficacy of green tea extract on cognitive function and impulsivity in the elderly. The ethical approval number is #18-157 from our IRB, and the randomized controlled trial registration number is UMIN 000036331. It was approved by the Ethics Committee of The University of Tokyo and carried out following the Declaration of Helsinki and the Ethical Guidelines for Medical and Health Research Involving Human Subjects. Participants provided written informed consent. Based on the United Nations agreed cut-off for elderly age [53], we recruited clinically normal adults over the age of 60 without diagnosis with dementia and MCI for the active and placebo groups. The study was designed to detect a 0.1 point difference in the Montreal Cognitive Assessment (MoCA) score at follow-up assuming a standard deviation (SD) of 0.18 with a type 1 error protection of 0.05 two-sided and 80% power. The number of necessary subjects was calculated to be 52. In total, 61 subjects were recruited at the beginning, and randomly divided into two groups: placebo and active. 5 subjects dropped out during the process, and 54 completed all tests (Placebo: *n* = 26, Active: *n* = 28). Of the 54 subjects, there were 15 men and 39 women, and the age was between 60 to 84 (Average: 73.6). Participants first underwent a cognitive psychological function test at the beginning of the trial, followed by a second cognitive psychological function test after 12 weeks of green tea extract consumption. Each subject received Matcha a green tea powder drink serving as 15 of g powder containing 1.5 g Matcha new green tea powder, Yabukita, (Kyoeiseicha Co., Ltd., Kyoto, Japan) with non-dairy creamer and low calories sweetener (ingredients are shown in Table 1), or placebo black tea flavored powder, twice a day for 12 weeks. In order to know the dietary habits of each subject, we also asked all of the participants to complete the Brief-type Self-administered Diet History Questionnaire (BDHQ) combined with a short Food Frequency Questionnaire (FFQ) for total polyphenol intake [54,55].

### 2.2. Cognitive and Memory Function Test

Cognitive function in participants was evaluated by MoCA [56] and Mini Mental State Examination (MMSE) [57]. Memory function was evaluated by the Wechsler Memory Scale-Delayed Recall (WMS-DR) [58], which belongs to a sub-category of the WMS-Revised. Higher scores in MMSE and MoCA mean better cognitive performances and higher scores in WMS-DR indicates better memory performance.

### 2.3. Assessment of Impulsivity

Impulsivity in participants was evaluated by the Barratt Impulsiveness Scale-11 (BIS-11) [59]. We used a Japanese version of BIS-11, which was confirmed to be reliable and valid [60]. BIS-11 is composed of 30 items describing common impulsive behaviors and preferences and each item is evaluated on a 4-point scale. Higher score in BIS-11 means elevated impulsivity. The original version of BIS-11 can be found in this reference [61].

### 2.4. Assessment of Dietary Habits

Since food culture and dietary habits vary by country, the questionnaires should be specifically chosen for each country. Thus, we chose the Brief Self-administered Diet History Questionnaire developed by Kobayashi et al. [54], which is widely used in assessing dietary habits in Japan, and estimated the nutrient intake based on the nutrient Standard Tables of Food Composition in Japan [62]. Each of the participants was asked to complete the questionnaire at the baseline, and the average frequency of eating and typical portion sizes in daily life were calculated.

### 2.5. Statistical Analysis

In order to investigate the effect of daily Matcha green tea powder supplementation on memory and impulsivity, we performed a two-way analysis of treatment × time interaction. A *p*-value of less than 0.05 was defined as statistically significant. In the analysis for each cognitive domain of MoCA in the female subgroup, multiple comparison correction was done by Bonferroni correction, and a *p* value of less than 0.0071 was defined as statistically significant. Besides, in order to assess the connection between a dietary habit and cognitive function we performed multiple regression analysis using the amount of change in the MOCA score as the objective variable and consumption of total polyphenols and each fat-soluble vitamin excluding test drinks as explanatory variables. Multiple regression analysis was performed individually for each gender. The items used as candidates for covariates were age, sex, body mass index, and years of education. The explanatory variables were included in the subsequent stage of analysis if the statistical significance was *p* ≤ 0.20 for the primary outcome. If a variable remained statistically significant at a level of *p* ≤ 0.20, it was incorporated into the final multivariable model. A Variance Inflation Factor (VIF) value over 10 is considered problematic for multicollinearity [63]. We determined that there was no multicollinearity between the explanatory variables since no factor had a VIF above 10. Microsoft Excel Add-in was used as a data analytic tool in this study. 

## 3. Results

### 3.1. Characteristics of Participants

Of the 61 participants, 54 completed all trials and were used for statistical analysis. 28 were assigned to the active group, and 26 to the placebo group. General characteristics are shown in Table 2. The Body Mass Index (BMI) ranged from 15.6 to 28.0 kg × m^−2^ (Average; 22.1 kg × m^−2^), and years of education ranged from 9 to 16 years (Average; 13.2). No significant differences were found in age, years of education, BMI, and gender ratio between the active and placebo groups (Table 2).

### 3.2. Primary Analysis

There were no significant changes in cognitive test scores (MoCA and MMSE) before and after the experiment, between the active and placebo groups. Likewise, no significant change was seen in memory test (WMS-DR) and the measurement of impulsivity (BIS-11) between the active and placebo groups (Table 3).

### 3.3. Secondary Analysis for Gender Subgroup

In the female subgroup, the analysis of MoCA scores showed significant improvement in the active subjects, compared to the placebo subjects (Treatment × Time Interaction, *p* = 0.0103; Table 4). The change of the MoCA score in the female subgroup was 1.95 ± 2.12 for the active subjects, and 0.15 ± 2.03 for the placebo subjects (Table 4). However, there was no significant change in the MMSE scores, between the active and placebo groups (Table 4). There was also no significant change in memory tests (WMS-DR) between the active and placebo groups (Table 4). Likewise, no significant difference was found in the changes in impulsivity measured by BIS-11 between the active and placebo groups (Table 4). As we detected the significant effect of green tea extract on MoCA score in the female subgroup, we conducted the analysis for each cognitive domain of MoCA, aiming to investigate the changes in cognitive function. The result showed that the MoCA score of the language domain showed significant improvement in the active subjects, compared to the placebo subjects (Treatment × Time Interaction, *p* = 0.0008; Table 5).

On the other hand, in the male subgroup, there was no significant change in the cognitive tests (MoCA and MMSE) between the active and placebo groups (Table 6). There was no significant change also in memory function (WMS-DR) between the active and placebo groups (Table 6). Likewise, no significant change was found in impulsivity measured by BIS-11 between the active and placebo groups (Table 6).

### 3.4. Multiple Regression Analysis of the Change Score for MoCA with the Amount of Daily Nutrient Intake, Excluding Test Drinks 

In order to assess the connection between dietary habit and cognitive function, we conducted a multiple regression analysis to determine the independent relationship between daily nutrient intake excluding test drinks and the score difference in MoCA for female subjects. As a result, we found that the higher consumption of vitamin K in daily diet excluding test drinks exhibited a significant inverse correlation with the total increasing score in MoCA (Table 7). In other words, it could be assumed that the beneficial effect on daily supplementation of Matcha may be more profound in people who had lower intake of vitamin K in daily life. Thus, we hypothesized that sufficient daily intake of vitamin K may be a crucial role related to cognitive function, as the vitamin K in Matcha green tea may act as a key component that maintains total daily intake to a sufficient level, and benefits cognitive function. Although total polyphenol intake excluding test drinks did not show any significant correlation with MoCA score (Table 8), the independent effect of tea catechin (EGCG), the main polyphenol inside tea, still needs to be confirmed, and other types of food questionnaire investigation may also be needed. 

## 4. Discussion

In this study, we conducted a randomized, double-blind, placebo-controlled 12-week trial using a psychometric test battery to investigate the effects of daily Matcha green tea powder supplementation on cognitive memory function and impulsivity in clinically normal elderly. As a result, improvement in cognitive function was seen in female subjects. This was shown in the difference in MoCA score changes from the start-up to follow-up between groups (*p* = 0.0103; +1.95 in the active group and +0.15 in the placebo). Especially in the language domain of the MoCA score, a noteworthy gain was seen in female subjects. We did not see any improvement in male participants. This female-specific result is consistent with previous studies showing that large-dose supplementation of decaffeinated green tea extract enhances working memory capacity of healthy elderly women [64]. 

In fact, gender difference in the clinical phenotype and progression of AD have been documented [65]. According to statistical analysis, women in the age range of 65–75 may have a higher increased risk of AD than men [66]. Mechanisms that underlie the gender difference may be associated with physiological changes that accompany estrogen loss and menopause [66]. On the other hand, it was reported that elderly women have a greater resilience to age-related cognitive decline compared with their male counterparts [67]. Besides, older women are known to have better reasoning and verbal abilities than older men and to perform better on verbal memory tests [68,69]. The gender difference in cognitive decline is also more pronounced in individuals who are cognitively normal before aging [67]. Collectively, these findings showed gender-specific traits. In our present study, we found a similar result in the MoCA score, which showed that the efficacy of Matcha green tea powder on cognitive function of elderly women was more pronounced in women rather than men, especially in the cognitive domain of language. 

As previous evidence showed, Matcha green tea was indicated as having a potential functional effect on cognitive function among middle aged and older subjects [70]. Furthermore, numerous nutrients inside green tea may pose beneficial effects on psychopathological symptoms by relieving stress and anxiety, and also may prevent stress-induced brain atrophy by modifying early stress response in animal studies [44,71]. In our present study, we also found a result which is consistent with these previous studies, again illustrating the possible neuro-modulatory effects of green tea.

MMSE is a widely used cognitive screening test, but to address the shortcomings of MMSE for detecting MCI, MoCA, which tests higher-level language and executive function, was developed as more challenging than MMSE [72]. MoCA has been reported to be more sensitive for detecting mild levels of cognitive impairment in AD and MCI [73,74], and it may be the reason why in our study MoCA was able to detect a cognitive change in higher level language function in the green tea extract supplementation group. Based on these findings, we concluded that the cognitive protective effect of matcha powder was detected by MoCA, which is a more sensitive cognitive function test [73,74], only in elderly women, who have greater resilience to age related cognitive decline [67], especially in the language domain, which is a cognitive function showing large gender differences [68,69]. However, in this study, the number of male subjects was much smaller than that of female subjects, and only 15 male subjects were used in the analysis in total. This may be the reason why no positive effect of matcha was observed on the cognitive function of male subjects. Further studies with appropriate sample sizes of male subjects would help to understand the gender-specific effect of matcha on cognitive function of elderly people.

However, no significant change in Delayed Recall (WMS-DR) scores was observed. This test is in a subordinate category of WMS and consists of the immediate and delayed recall of a verbally-presented story containing meaningful information [75]. We performed this to evaluate the episodic memory function of our participants. To the best of our knowledge, no long-term effect on episodic memory from continuous consumption of tea or tea extracts has been reported. Therefore, we concluded that consumption of green tea extract was not linked with the enhancement of chronic episodic memory function.

As mentioned above, the cognitive tests used in this study were two screening tests for dementia and a logical memory delayed recall test. There are various types of test that measure cognitive ability, and there are tests that measure more advanced and complex cognitive functions. Further results may have been observed by performing these additional tests. However, the purpose of this study was to establish a method of intervention in daily life to reduce the incidence of AD by adjusting nutrition. Therefore, we focused on overall cognitive function which declines with dementia, and episodic memory function, which is impaired especially in AD, rather than advanced cognitive function [76].

Matcha green tea powder contains several active compounds, including tea catechin, EGCG, and other active compounds such as caffeine and theanine, which have been recognized as functional compounds related to cognitive function. EGCG crosses the blood brain barrier [77], and exerts neuroprotective effects against amyloid *β*(A*β*) toxicity by inhibiting A*β* aggregation [78] and production [79], while L-theanine also crosses the blood brain barrier and ameliorates mood [80,81]. Due to its structural analogy with glutamate, the principal excitatory neurotransmitter in the brain, L-theanine could cause a favorable downshift in neurodegeneration [82]. Furthermore, L-theanine and caffeine are known to improve cognitive performance, either by increasing the dopaminergic and cholinergic transmission in the brain, or through glutamatergic mechanisms. The previous study showed that the combination of these compounds caused significant improvement in cognitive performance, possibly due to the modulation of neuronal activities in the brain [45]. It has also been suggested that if taken together these compounds have additive effects on improving attention span [83]. Although the independent functional effect among these active compounds has not yet been well elucidated, EGCG, L-theanine, and caffeine may either function in a separate way or in a collaborative way. In this study, total dietary polyphenol intake did not show a significant correlation with MoCA scores. However, due to the fact that the estimation of nutrient intake in BDHQ was based on the Standard Tables of Food Composition in Japan, we could only elucidate the relationship with total daily intake of polyphenol, as independent estimation of each category of polyphenol, like EGCG, is limited. Thus, it is a limitation that we cannot successfully capture the specific effect of EGCG in daily life in our present study via BDHQ. Besides, BDHQ does not include matcha green tea. Matcha green tea is an expensive drink even in Japan, and although participants were unlikely to be consuming it daily before the study, it cannot be ruled out that some participants were already consuming it. Therefore, the intervention did not increase consumption in some participants and this could have affected results observed.

It should be noticed that due to the special processing of Matcha green tea, it presents higher amounts of fat-soluble nutrients than regular green tea, especially vitamin K and lutein, both of which show a beneficial effect on cognitive function in either animal or epidemiological studies [49,51,84,85]. Notably, in our dietary analysis, we found that higher consumption of vitamin K in daily diet excluding test drinks was inversely correlated with an increase in the MoCA score, which suggested the role of supplementing deficient vitamin K for the amelioration of cognitive deficits. Although the direct mechanism between vitamin K and cognitive function has not yet been fully elucidated, it was reported that lifetime low consumption of vitamin K resulted in a mild cognitive impairment in aged rats, and a similar pattern was found in early stage of probable Alzheimer’s disease subjects [84]. Yet a positive association between serum phylloquinone and performance in verbal episodic memory was reported, which may suggest that daily supplementation of vitamin K can pose benefits in cognitive function [48]. In our present study, we found vitamin K exists in higher concentration in Matcha green tea powder than in the placebo, as taking two cups of Matcha green tea per day elevates vitamin K daily intake by 60% in all subjects [86]. As discussed above, there is a significant change in the MoCA score in that female subgroup that were supplemented with Matcha green tea powder, which contains Vitamin K. Interestingly, the concentration of Menaquinone-4, the prevalent form of vitamin K in the brain, has been shown to be higher in female than in male rats [48]. If this were to be also applicable to humans, one could argue that the female brain absorbs dietary vitamin K more than the male, possibly due to the gender difference in metabolism, and that is why the effect of vitamin K intake was more easily seen in the female subgroup in our experiment. Taken together, it may be suggested that vitamin K in Matcha green tea powder acts as an active nutrient that could reduce deterioration of cognitive function. Although the analysis of the independent effect each compound could have on cognitive function is still necessary, these findings support the idea that the consumption of green tea improves cognitive performance, as we have demonstrated.

On the other hand, we found that higher consumption of vitamin E in daily diet excluding test drinks was correlated with an increase in the MoCA score, which may suggest that the higher the daily intake of vitamin E, the greater the effect of matcha green tea powder intake on cognitive function. Green tea polyphenols are known to exhibit synergistic antioxidant activity with one of the vitamin E α-tocopherol in micelles, homogeneous solutions and in human low density lipoproteins [87]. Combination therapy of vitamin E and green tea polyphenols shows synergistic protective effect against myocardial infarction and diabetes in rats [88,89]. So far, no combined protective effect of vitamin E and green tea ingredients on cognitive function has been reported, but these facts motivate us to do further research investigating the combined effects of matcha green tea powder and vitamin E on cognitive function. The matcha powder used in this study contains 89 μg of vitamin K and 0.8 mg of vitamin E per day, which corresponds to 59% and 12% of the daily intake standard set by the Japanese Ministry of Health, Labor and Welfare [90]. Based on these facts, we considered that the reason why the improvement effect on MoCA was greater when the intake of vitamin K was low in the daily diet excluding matcha was that the intake of matcha was able to supplement most of the vitamin K deficiency; whereas, since the amount of vitamin E contained in matcha is relatively low, it seems that the reason why the improvement effect of MoCA was large when the intake of vitamin E was low was not because the intake of matcha supplemented the deficiency of vitamin E, but because of the combined effect of vitamin E and green tea polyphenols.

In this study, the amount of matcha powder consumed per day was 3 g. In previous clinical trials investigating the non-acute effects of green tea powder on cognitive function, consumption was similar to that in this study (2 g/day [91]; 4 g/day [45]). The drinks in the active group contained 81 mg of caffeine per day. The average consumption of caffeine per day in the elderly is about 200 mg, and the responses to caffeine in some physiological systems are known to be greater in the elderly at doses in the 200 to 300 mg range [92]. High caffeine intake can cause acute caffeine poisoning. Besides, routine moderate caffeine intake causes addiction and withdrawal symptoms, for example, when an average daily intake of 235 mg is interrupted, 52% will experience headaches two days later [93]. In addition, the matcha drink used in this study is a rich and strong drink, and it is not realistic to take such a drink frequently on a daily basis. Therefore, we considered that the amount and frequency of matcha intake in this study was appropriate.

No significant effect of Matcha green tea powder on impulsive changes was observed in either primary analysis or gender subgroup analysis. Impulsivity is a polysemous concept but is typically defined as a predisposition toward rapid, unplanned reactions to internal or external stimuli, in spite of negative consequences these impulsive actions could bring to oneself and others [30]. Besides, impulsivity is recognized as an intermediate phenotype of various behavioral abnormalities, and can be classified as having a multidimensional structure, including actions without an appropriate amount of deliberation [94], risk-taking behaviors [95], and the tendency to choose immediate rewards over long-term rewards [96]. Impulsivity is a component of personality as well and is often assessed by personality tests, and BIS-11 is one of the most widely used personality tests for impulsivity [97]. In the previous study, it was shown that the ingestion of green tea components has a beneficial effect on mood, by reducing stress and anxiety [36,98]. Therefore, in this study also we have investigated the effect of Matcha green tea powder on impulsivity, but our study did not show any strong correlation between its consumption and reduction in impulsivity. However, unlike mood, personality does not change quickly, and therefore the effects may not have been observed in as little as 12 weeks. In future studies we may conduct to investigate the effect of Matcha green tea powder on impulsivity, we need to focus on impulsivity as a behavioral paradigm measured by behavioral tests, such as go/no-go tasks and delayed discounting tasks [99].

Our study has some limitations. First, the sample size adopted may not have been sufficient enough to detect differences in scores of the cognitive and psychometric tests performed. Especially in MoCA, larger sample sizes might have been able to detect differences in the scores of total subjects. Second, there was a concern about translation in the impulsivity assessment test. Language nuance is an important factor in translation, so that the intent of the questioner can be accurately communicated, as the BIS-11 is in a questionnaire format. However, there are some items for which internal consistency has not been established in the Japanese version of BIS-11 [60]. This may be one of the reasons why the effect of green tea extract on impulsive changes was not detected. Further studies with adequate sample sizes and linguistically optimized tests could reveal much more pronounced results.

## 5. Conclusions

This is the first report to investigate the effects of daily Matcha green tea powder supplementation on cognitive and memory functions and impulsivity in clinically normal elderly individuals. In summary, no significant effect of Matcha green tea powder was shown in total subjects during the trial, but a significant effect on cognitive changes, especially in language domain, was shown in female subjects. Our results suggest that daily supplementation of Matcha green tea powder may have a beneficial effect against cognitive decline in clinically normal elderly women. Though we could not know for sure which is the most fundamental bioactive compound in Matcha green tea powder that enhances cognition, hydrophobic compounds such as vitamin K may be considered a possible candidate. We shall investigate the independent activities of each compound and underlying mechanisms in future studies. 

## Figures and Tables

**Table 1:** Composition of the Active drink with Matcha green tea powder and placebo drink.

Content	Active	Placebo
Energy (kcal)	64	64
Protein (g)	0.8	0.4
Fat (g)	1.7	1.7
Carbohydrate (g)	11.6	12.2
Sodium (mg)	36.6	38.5
Vitamin C (mg)	2.1	0.0
Vitamin K (μg)	44.4	2.0
Total Polyphenol (mg)	160.5	31.5
Tea catechin (mg)	138.5	2.5
Lutein (mg)	1.087	0
L-theanine (mg)	25.2	0.0
Caffeine (mg)	40.5	2.4

The data are nutritional information on powdered drinks supplied at 15 g per serving, where active contains 1.5 g of Matcha green tea powder. Subjects consumed two servings per day for the intervention.

**Table 2:** Participant Characteristics

Item	Active	Placebo	*p*-Value
Age	74.4 ± 5.6 ^a^	73.0 ± 5.3 ^a^	0.36 ^c^
Gender (M/F)	9/19 ^b^	6/20 ^b^	0.57 ^d^
BMI	22.4 ± 2.8 ^a^	21.8 ± 2.8 ^a^	0.48 ^c^
Years of Education	13.8 ± 1.7 ^a^	12.9 ± 2.1 ^a^	0.09 ^c^

The data are for 54 participants who completed all trials. ^a^ Mean ± Standard Deviation; ^b^ Number of people; ^c^ *p*-Value was determined by the student’s *t*-test; ^d^ *p*-Value was by the chi-square test BMI—body mass index; M—male; F—female.

**Table 3:** Two-way analysis of treatment × time interaction in psychometric tests of active and placebo groups for total subjects.

Test	Start-Up		Follow-Up		Treatment × Time Interaction		
	Active ^a^	Placebo ^a^	Active ^a^	Placebo ^a^	Active ^a^	Placebo ^a^	*p*-Value ^b^
Montreal Cognitive Assessment (MoCA)	25.1 ± 2.5	26.3 ± 2.7	26.6 ± 2.5	26.7 ± 2.2	1.6 ± 2.0	0.6 ± 2.0	0.0859
Mini-Mental State Examination (MMSE)	27.1 ± 2.2	27.5 ± 1.4	27.8 ± 2.0	28.1 ± 1.2	0.7 ± 2.1	0.7 ± 1.9	0.9643
Wechsler Memory Scale—Delayed Recall (WMS-DR)	7.4 ± 3.4	5.8 ± 3.8	9.7 ± 0.7	8.0 ± 4.0	2.3 ± 2.9	2.2 ± 2.5	0.8984
Barratt Impulsiveness Scale-11 (BIS-11)	52.6 ± 6.1	55.2 ± 6.6	52.8 ± 7.5	54.6 ± 6.1	0.2 ± 5.6	−0.5 ± 5.7	0.7652

^a^ Mean ± Standard Deviation; ^b^ Changes between start-up and follow-up.

**Table 4:** Two-way analysis of treatment × time interaction in psychometric tests of active and placebo groups for female subjects.

Test	Start-Up		Follow-Up		Treatment × Time Interaction		
	Active ^a^	Placebo ^a^	Active ^a^	Placebo ^a^	Active ^a^	Placebo ^a^	*p*-Value ^b^
MoCA	24.9 ± 2.2	26.8 ± 2.3	26.8 ± 2.4	26.9 ± 1.9	1.9 ±2.1	0.2 ± 2.0	0.0103 *
MMSE	26.9 ± 2.1	27.7 ± 1.4	28.1 ± 1.4	28.3 ± 1.2	1.1 ±2.3	0.6 ± 2.0	0.5167
WMS-DR	7.0 ± 2.8	5.6 ± 3.7	10.0 ± 3.7	7.6 ± 3.6	3.0 ± 2.7	2.0 ± 2.3	0.2204
BIS-11	61.6 ± 5.9	64.3 ± 6.0	60.2 ± 10.0	64.1 ± 5.3	−1.4 ± 5.1	−0.2 ± 4.7	0.5112

^a^ Mean ± Standard Deviation; ^b^ Changes between start-up and follow-up. * *p* < 0.05.

**Table 5:** Two-way analysis of treatment × time interaction in each cognitive domain scores of MoCA of active and placebo groups for female subjects.

Sub-Category	Start-Up		Follow-Up		Treatment × Time Interaction		
	Active ^a^	Placebo ^a^	Active ^a^	Placebo ^a^	Active ^a^	Placebo ^a^	*p*-Value ^b^
Executive	4.6 ± 0.6	4.5 ± 0.9	4.6 ± 0.7	4.7 ± 0.5	−0.1 ±0.9	0.3 ±0.8	0.2733
Naming	3.0 ± 0.0	2.9 ± 0.2	3.0 ± 0.0	3.0 ± 0.0	0.0 ± 0.0	0.1 ± 0.2	0.3364
Attention	5.3 ± 0.9	5.9 ± 0.4	5.5 ± 0.8	5.9 ± 0.5	0.2 ± 1.0	0.0 ± 0.6	0.5315
Language	1.3 ± 0.7	2.1 ± 0.9	2.0 ± 0.9	1.8 ± 0.7	0.7 ± 0.9	−0.3 ± 0.9	0.0008 **
Abstraction	1.8 ± 0.4	2.0 ± 0.2	2.0 ± 0.0	2.0 ± 0.0	0.2 ± 0.4	0.1 ± 0.2	0.1411
Delayed Recall	3.2 ± 1.5	3.4 ± 1.5	3.7 ± 1.3	3.3 ± 1.4	0.6 ± 1.3	−0.1 ± 1.6	0.1640
Orientation	5.1 ± 0.6	5.5 ± 0.5	5.5 ± 0.5	5.7 ± 0.5	0.4 ± 0.8	0.2 ± 0.8	0.5146

^a^ Mean ± Standard Deviation; ^b^ Changes between start-up and follow-up. Multiple comparison correction was d by Bonferroni correction, and a *p* value of less than 0.0071 was defined as statistically significant. ** *p* < 0.0014.

**Table 6:** Two-way analysis of treatment × time interaction in psychometric tests of active and placebo groups for male subjects.

Test	Start-Up		Follow-Up		Treatment × Time Interaction		
	Active ^a^	Placebo ^a^	Active ^a^	Placebo ^a^	Active ^a^	Placebo ^a^	*p*-Value ^b^
MoCA	25.4 ± 2.9	23.4 ± 2.4	25.8 ± 3.0	26.4 ± 3.3	0.4 ± 1.8	3.0 ± 2.4	0.0659
MMSE	27.3 ± 2.5	26.9 ± 1.2	26.8 ± 3.2	27.9 ± 1.5	−0.5 ± 1.4	1.0 ± 1.8	0.2620
WMS-DR	7.9 ± 4.5	6.7 ± 3.9	9.0 ± 3.6	9.7 ± 4.8	1.1 ± 2.8	3.0 ± 2.8	0.1960
BIS-11	55.4 ± 5.4	57.3 ± 7.1	58.3 ± 6.8	55.3 ± 8.6	2.9 ± 5.6	−0.5 ± 8.1	0.1270

^a^ Mean ± Standard Deviation; ^b^ Changes between start-up and follow-up.

**Table 7:** Multiple Regression Analysis of the change in MoCA score and the number of fat-soluble vitamins.

Variable	*B*	SE *B*	*β*	F Value	t Value	*p*-Value
Active/Placebo(1/0)	1.5098	0.6975	0.3410	4.9370	2.2291	0.0333 *
Age	−0.0921	0.0626	−0.2208	2.1691	−1.4728	0.1503
Vitamin D	−0.0584	0.0357	−0.3974	2.6797	−1.6370	0.1111
Vitamin E	0.5249	0.2186	0.9092	5.7673	2.4015	0.0221 *
Vitamin K	−0.0061	0.0025	−0.6508	5.7432	−2.3965	0.0224 *

Among the fat-soluble vitamins, vitamin A was deviated from variables during the sequential multiple regression analysis with number of covariates, including body height and weight. *B* represents a partial regression coefficient. SE *B* represents standard error of the partial regression coefficient. *β* represents standardized partial regression coefficient. * *p* < 0.05.

**Table 8:** Multiple Regression Analysis of the change score of MoCA with the amount of daily intake of polyphenol.

Variable	*B*	SE *B*	*β*	F Value	t Value	*p*-Value
Active/Placebo(1/0)	1.8900	0.7178	0.4269	6.9326	2.6330	0.0128
Age	−0.0790	0.0656	−0.1893	1.4496	−1.2040	0.2372
Total Polyphenol	−0.1846	1.3636	−0.0210	0.0183	−0.1354	0.8931

*B* represents a partial regression coefficient. SE *B* represents a standard error of the partial regression coefficient. *β* represents a standardized partial regression coefficient.
